# A Variational Quantum Linear Solver Application to Discrete Finite-Element Methods

**DOI:** 10.3390/e25040580

**Published:** 2023-03-28

**Authors:** Corey Jason Trahan, Mark Loveland, Noah Davis, Elizabeth Ellison

**Affiliations:** 1 Information and Technology Laboratory, U.S. Army Engineer Research and Development Center, Vicksburg, MS 39180, USA; 2 Applied Research Laboratories, The University of Texas at Austin, Austin, TX 78713, USA

**Keywords:** quantum computing, quantum variational algorithm, finite-element methods, Poisson equation, heat equation, quantum algorithms

## Abstract

Finite-element methods are industry standards for finding numerical solutions to partial differential equations. However, the application scale remains pivotal to the practical use of these methods, even for modern-day supercomputers. Large, multi-scale applications, for example, can be limited by their requirement of prohibitively large linear system solutions. It is therefore worthwhile to investigate whether near-term quantum algorithms have the potential for offering any kind of advantage over classical linear solvers. In this study, we investigate the recently proposed variational quantum linear solver (VQLS) for discrete solutions to partial differential equations. This method was found to scale polylogarithmically with the linear system size, and the method can be implemented using shallow quantum circuits on noisy intermediate-scale quantum (NISQ) computers. Herein, we utilize the hybrid VQLS to solve both the steady Poisson equation and the time-dependent heat and wave equations.

## 1. Introduction

Quantum computing has reached a new era where theory is transitioning into practice as quantum computers and simulators become more widespread and available to the scientific community. This transition has encouraged algorithmic exploration, with an intent toward showing “quantum supremacy” or “quantum advantage”. Quantum advantage refers to the demonstrated and measured success in processing a real-world problem faster on a quantum computer than on a classic computer. Quantum supremacy [1], on the other hand, refers to the demonstrated and measured ability to process **any problem** faster on a quantum computer, regardless of its real-world applicability [2].

In 2019, Arute et al. [3] claimed to have achieved quantum supremacy using a programmable superconducting processor by “performing a series of operations in 200 s that would take a supercomputer about 10,000 years to complete”. In December 2020, a group based out of the University of Science and Technology of China (USTC) led by Jian-Wei Pan claimed quantum supremacy by implementing Gaussian boson sampling on 76 photons with their photonic quantum computer [4]. The paper states that to generate the number of samples the quantum computer generates in 20 s, a classical supercomputer would require 600 million years of computation. Although these supremacy claims have been the source of much recent debate, mostly with respect to whether or not their classical comparisons are the most efficient, it is clear that we are on the threshold of a new age of computation, heralded by today’s noisy intermediate-scale quantum (NISQ) hardware.

Today’s NISQ computers are limited in scalability because they are (1) subject to noise and thus not fault-tolerant, and (2) they are qubit-limited (usually meaning less than 100 qubits). Regarding the latter, however, the number of qubits on modern day quantum computers is rapidly growing, with IBM projecting a remarkable 1121 qubit system in 2023. Although this exponential qubit growth is vital in the near term, “shot” noise arising form the Heisenberg uncertainty principle and zero-point thermal fluctuations cause a phenomenon called decoherence, which may ultimately prevent scalability to larger qubit applications. Quantum systems achieve their notable advantage over classical ones via entanglement, a process by which a pure state quantum system develops a probability distribution over multiple classical outcomes. Entanglement gives quantum computers the ability to process and store exponentially more information than a classical computer. Noise, however, introduces errors that cause decoherence in the entanglement and can significantly degrade the performance of NISQ computers [5,6,7,8]. In fact, much of today’s quantum computing efforts are in noise mitigation [9,10,11,12,13,14]. In January 2022, for example, a group of scientists from the University of Chicago and Purdue University collaborated on a new promising noise control technique: Instead of directly trying to measure the noise, they constructed a unique “fingerprint” of the noise on a quantum computer as it was seen by a program run on the computer [15]. This approach shows promise for mitigating the noise problem, as well as suggesting ways that users could actually turn noise into an advantage.

Despite these drawbacks, NISQ computers remain promising in application areas such as quantum chemistry, cybersecurity, drug development, financial modeling, traffic optimization, weather forecasting, climate change prediction, artificial intelligence and machine learning. Over the last few years, quantum hardware has become available to the average researcher, mostly through two types of cloud computing. The first type is cloud services providing access to a single company’s collection of quantum devices. The Qiskit cloud service offered by IBM Quantum [16] is the premier example of this. On the other hand, there are multi-platform services such as Amazon Braket [17] that work as intermediaries to give users options to access quantum devices owned by multiple vendors. In most cases, cloud computing interfaces for quantum devices are implemented in Python to provide starting points for accessing working quantum devices. Introductory resources for algorithmic understanding and design are also widely available to the public. For example, the IBM Qiskit textbook [18] provides a college-level introduction to quantum information with integrated programming exercises, the Codebook by Xanadu [19] provides an introductory course built around the Pennylane package, allowing for differentiable programming of quantum computers, and QBraid is an online platform for developing quantum software with introductory quantum tutorials [20].

As near-term supremacy does not mean utility, many today utilize these current cloud resources for the investigation of quantum advantages for practical problems. NISQ computers must be restricted to “shallow” circuits for noise control. These circuits have a minimal number of qubits that are more easily controlled. One way of keeping quantum circuits shallow, for example, is by combining quantum and classical algorithms so that only the computationally intensive portion of the problem is implemented on the quantum computer, thereby offering some degree of quantum speed-up or advantage while maintaining shallow circuits amenable to NISQ computers. This type of hybrid set-up is somewhat analogous, for example, to classical GPU acceleration. Recently, hybrid methods such as these have been utilized for near-term acceleration of machine learning and optimization problems [21,22,23,24,25,26,27,28,29]. A number of quantum algorithms for machine learning are based on the idea of amplitude encoding, which associates the amplitudes of a quantum state with the inputs and outputs of computations [24,30,31]. Since a state of *m* qubits is described by $2^{m}$ complex amplitudes, this information encoding can allow for an exponentially compact representation. Intuitively, this corresponds to associating a discrete probability distribution over binary random variables with a classical vector. The goal of algorithms based on amplitude encoding is to formulate quantum algorithms whose resources grow polynomially in the number of qubits *m*, which amounts to a logarithmic time complexity in the number of amplitudes and therefore the dimension of the input.

Many quantum machine learning algorithms are based on variations in the quantum algorithm for linear systems of equations [32] (colloquially called HHL after the paper’s authors) which, under specific conditions, perform a matrix inversion using an amount of physical resources growing only logarithmically in the dimensions of the matrix. One of these conditions is that a Hamiltonian which, entry-wise, corresponds to the matrix can be simulated efficiently, which is known to be possible if the matrix is sparse [33] or low in rank [23]. Quantum matrix inversion can be applied to machine learning methods in which the training reduces to solving a linear system of equations, such as in least squares linear regression [30,31], the least squares version of support vector machines [24], and Gaussian processes [34].

For suitably conditioned linear systems, the HHL algorithm scales logarithmically in *n*, suggesting the possibility of exponential speed-up over classical systems [32], which holds promise for quantum computers beyond the NISQ era. In today’s NISQ machines, however, shot noise has dramatically limited the size of the linear systems directly solvable by the HHL algorithm. To date, 2×2 systems have been solved by superconducting qubits [35,36], nuclear magnetic resonance [37], and photonic devices [38,39]. The largest system solved on a gate-based computer was an 8×8 problem using NMR [40].

Given today’s NISQ limitations of the HHL algorithm, an alternative method for linear system solution has been proposed to gain a quantum advantage: variational hybrid quantum-classical algorithms (VHQCAs). VHQCAs are capable of providing an advantage to Shor’s algorithm for factoring [41] and have gained momentum in the fields of chemistry [42,43,44,45], simulation [46,47,48,49,50], data compression [51], state diagonalization [52,53,54], compiling [55,56], quantum foundations [57], fidelity estimation [58], and meteorology [59]. The general VHQCA algorithm reduces the quantum circuit depth by using a classical optimizer and only evaluating the cost/objective function on the quantum computer.

In this study, we continue to investigate quantum advantages in classical problems by utilizing a VHQCA recently introduced by Bravo-Prieto et al. [60,61] called the variational quantum linear solver (VQLS) to obtain finite-element solutions to the Poisson, heat, and wave equations. The quantum/classical hybrid VQLS is a method for solving linear systems on near-term quantum computers which variationally prepares a quantum state |x〉 such that A|x〉∝|b〉. Bravo-Prieto et al. were able to derive a meaningful termination condition for VQLS that allows one to guarantee a desired solution precision with efficient quantum circuits to estimate the variational cost function *C* while providing evidence for the classical hardness of its estimation. Using Rigetti’s quantum computer, the VQLS was used for solutions up to a problem size of 1024×1024 (10 qubits), which is the largest implementation of a linear system on quantum hardware to date. The time complexity of the VQLS was heuristically found to scale efficiently with the linear solution precision ϵ, the matrix condition number κ, and the linear system size *N*.

## 2. The Variational Quantum Linear Solver

The quantum/classical hybrid VQLS [60,61] algorithm attempts to find a solution to the linear system such that A|x〉∝|b〉 by minimizing a scalar cost function based on the scaled projection of A|x〉 onto |b〉. The solution vector |x〉 is approximated with a wave function created through a quantum circuit ansatz. To prepare a linear system for VQLS solution, the matrix A must be expressed as a linear combination of universal quantum gates. Additionally, the right-hand side (RHS) of the linear system must be transformed into a normalized quantum state |b〉, which can be generated by unitary operations *U* applied to the ground state of some number of qubits. We now discuss these elements of the VQLS in detail.

### 2.1. The Variational Ansatz

In the VQLS algorithm, |x〉 is prepared by acting on the |0〉 state with a trainable gate sequence V(α). The ansatz V(α) can be expressed in terms of *L* gates from a gate alphabet $A=G_{k}\left(\alpha \right)$ as

(1) $$\begin{array}{c} V\left(\alpha \right)=G_{k_{L}}\left(\alpha_{L}\right)\cdots G_{k_{i}}\left(\alpha_{i}\right)\cdots G_{k_{1}}\left(\alpha_{1}\right) \end{array}$$

Here, $\vec{k}=\left(k_{L},\cdots ,k_{1}\right)$ identifies the types of gates and their placement in the circuit (i.e., on which qubit they act), while α represents the continuous parameters over which optimization occurs. All results presented herein are based on a “fixed ansatz”, where $\vec{k}$ is fixed over time and *V* is only optimized over α. Though it was not investigated in this study, variable ansatz optimization was shown to improve convergence in some cases in [52,62].

**Figure 1:**
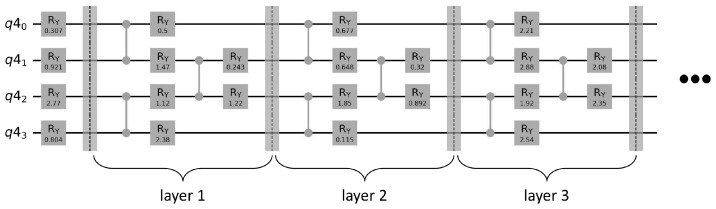
A four-qubit example of the fixed ansatz used for this study.

Training of the ansatz is performed layer by layer, just as in neural networks. The number of layers is decided by the user. Although the solution function space widens as the layers are increased, over-determined parameter optimization may become difficult and inefficient. The properties of a good ansatz are as follows: (1) the circuit is shallow, minimizing decoherence, (2) it has minimal optimization parameters, and (3) the ansatz should span the space where the solution lives. Of all the layer structures we tested, the ansatz given in [60] (shown in Figure 1) was the most optimal one. This ansatz begins with an initial y rotation (Ry) of each qubit before moving on to the layered portion of the circuit.

Each layer starts with alternating controlled-z (CZ) gates followed by Ry rotations on the controlled qubits. The CZ gates have the crucial function of entangling the qubits, which allows for an exponentially larger space representation than a purely classical cost evaluation. The Ry gates allow one to “search” the state space by varying the rotational parameters.

In this study, a range of layers were tested for each application of the VQLS. Some general guidelines for choosing the number of layers were found: (1) a greater number of layers was needed, as the problem’s dimensionality was increased (resulting in larger linear systems), and (2) a greater number of layers was required, as the number of terms in the Pauli decomposition of the stiffness matrix grew. These two factors greatly limited the size of the finite-element problems we could test at this time to a maximum of 10 nodes (8 internal nodes or 3 qubits).

### 2.2. Matrix Pauli Decomposition

In order to solve the linear system using the VQLS, the matrix must be represented as a linear combination of Hermitian unitary operators $A=\sum_{i}c_{i}\;U_{i}$, representing a system Hamiltonian where $U_{i}$ represents the unitaries and $c_{i}$ represents complex coefficients. Additional assumptions are that the matrix condition number κ<inf and ∥A∥≤1 and that the $A_{i}$ unitaries can be implemented with efficient quantum circuits. Typically, this decomposition consists of a linear combination of Kronecker products of the Identity and Pauli matrices, as these gates are widely used and recognized. These matrices and gates are defined as follows:

(2) $$\begin{array}{c} I=\left(\begin{array}{cc} 1 & 0\\ 0 & 1 \end{array}\right),\;\;X=\left(\begin{array}{cc} 0 & 1\\ 1 & 0 \end{array}\right),\;\;Y=\left(\begin{array}{cc} 0 & -i\\ i & 0 \end{array}\right),\;\;Z=\left(\begin{array}{cc} 1 & 0\\ 0 & -1 \end{array}\right) \end{array}$$

For all application matrices herein, a recently proposed algorithm given in [63], which takes a square real symmetric matrix of an arbitrary size and decomposes it into a tensor product of Pauli spin matrices, was used. The routine was given by the authors in Python and is publicly available. The mathematical procedure for generating this decomposition for a general-sized stiffness matrix, often encountered in discrete finite-element methods, is given in Appendix A.

### 2.3. Right-Hand Side Preparation

The VQLS requires that the linear system RHS be transformed into a normalized quantum state |b〉 generated by some series of unitary operations U applied to the ground state of the qubits:

(3) |b〉=U|0〉

Again, we assume that U can be efficiently implemented with a quantum circuit. For example, if the boundary conditions are homogeneous, and a reduced linear system is used which includes only the internal domain grid points, then the constant RHS wave function can be created by a quantum circuit which applies a Hadamard gate to each qubit:

(4) $$|b\rangle =(H_{0}H_{1}H_{1}\cdots H_{m-1})|0\rangle$$

where *m* is the total number of qubits used to represent the reduced system. In general, however, the RHS vector of the linear systems will not be constant, and a vector-specific circuit must be generated. For the applications herein, we utilized the “isometry” package in Qiskit to produce the corresponding quantum state from a specific RHS vector. It is worth noting that more general, non-constant RHSs may lead to deeper, more complex circuitry that may affect the VQLS’s efficiency, since this circuit is evaluated in a controlled manner during each cost calculation.

## 3. Computational Details

The VQLS in this study was implemented in Python using IBM’s Qiskit [16]. Qiskit is an open source software development kit for working with OpenQASM and the IBM Q quantum processors. For prototypical applications, such as those needed for the early stages of this work, Qiskit offers a quantum computer simulator which allows the user to build and test quantum circuits on a local machine without the need for a quantum computer. The Qiskit package, along with its statevector simulator, can be imported into a Python script in the usual way. For all problems in this study, the Qiskit Aer simulator backend was used.

## 4. Training Algorithm

Scientific Python (SciPy) offers a variety of options for both constrained and unconstrained optimization of scalar objective/cost functions. The purpose of these optimizers is to update the parameters of the VQLS ansantz. Generally speaking, multi-variant objection function optimizers fall into two categories: gradient- and non-gradient-based optimization. Gradient-based methods, such as the Newton conjugate gradient method, use the objective function gradients (i.e., Jacobians or Hessians) to move in a descending direction toward a minima. Non-gradient methods, on the other hand, work by iteratively approximating the actual constrained optimization problem with linear programming problems. During an iteration, an approximating linear programming problem is solved to obtain a candidate for the optimal solution. The candidate solution is evaluated using the original objective and constraint functions, yielding a new data point in the optimization space. This information is used to improve the approximating linear programming problem used for the next iteration of the algorithm. When the solution cannot be improved anymore, the step size is reduced, refining the search. When the step size becomes sufficiently small, the algorithm finishes.

Previous studies have compared gradient- and non-gradient based optimization for a range of VQLS applications using quantum simulators, quantum simulators with shot noise, and fully quantum applications. In particular, in [64], it was shown that once shot noise is included in either the statevector simulator or real quantum application, gradient-based optimizers do not offer much of an advantage over non-gradient optimizers. A popular choice for VQLS applications, for example, is the non-gradient based constrained optimization by linear approximation (COBYLA) method. Due to these previous findings and the complexity of including the objective function gradients, the COBYLA method was used for all applications herein, and gradient-based methods were not investigated.

## 5. Applications

### 5.1. Application 1: The Poisson Equation

For the first application, the QVA was used to solve the Dirichlet problem for the 1D Poisson equation, given in strong form by

(5) $$\begin{array}{cc} -▵u(x) & =f(x),\;\;u(x)\in \Omega , \end{array}$$

where $u\left(0\right)=u_{L}$ and $u\left(1\right)=u_{R}$. The equivalent weak representation of this equation is obtained by taking Equation (5) and multiplying it by an arbitrary test function in the appropriate function space, followed by integrating by parts [65] to give

(6) $$\begin{array}{c} \int_{u_{L}}^{u_{R}}\frac{d\phi }{dx}\frac{du}{dx}dx=\int_{u_{L}}^{u_{R}}\phi f\left(x\right)\;dx\;\;\;\;\;\forall \phi \in H_{0}^{1}\left(\Omega \right) \end{array}$$

Here, ϕ(x) is the arbitrary test function in the appropriate Hilbert space, and the boundary term from integrating by parts vanishes since the test space $H_{0}^{1}$ has 0 trace. To discretize this equation, the standard Galerkin approximation with linear Lagrange polynomials is used on a uniform 1D grid of *N* points, where the *i*th nodal location is given by $x_{i}=ih$. Here, h=1/(N−1) and 0≤i≤(N−1). Additionally, we define n=N−2 as the internal node count. This discretization results in the linear system

(7) $$\begin{array}{c} K\vec{u}=\vec{f} \end{array}$$

where for linear, Lagrangian basis function support, K is the typical tridiagonal “stiffness” matrix, $\vec{u}$ is the solution vector, and $\vec{f}$ is the right-hand side. When applying non-homogeneous Dirichlet boundary conditions, it is essential to manipulate this linear system to force the specified solution values on the domain endpoints, giving the following RHS:

(8) $$\begin{array}{cc} \vec{f} & =\left(\begin{array}{c} \int_{0}^{1}\phi_{1}\;f\left(x\right)\;dx+\int_{0}^{1}\frac{\partial \phi_{1}}{\partial x}\frac{\partial \phi_{0}}{\partial x}\;u_{L}\;dx\\ \int_{0}^{1}\phi_{2}\;f\left(x\right)\;dx\\ \int_{0}^{1}\phi_{3}\;f\left(x\right)\;dx\\ \vdots \\ \int_{0}^{1}\phi_{n}\;f\left(x\right)\;dx+\int_{0}^{1}\frac{\partial \phi_{n}}{\partial x}\frac{\partial \phi_{n+1}}{\partial x}\;u_{R}dx \end{array}\right) \end{array}$$

For Dirichlet boundary conditions, a reduced system can be solved without the endpoints, since these are known. The reduced matrices were used for all applications herein to increase the grid resolution, since the qubit count was extremely limited. While obtaining the quantum wavefunction for the RHS of the homogeneous Poisson equation is relatively straightforward, heterogeneous boundaries or time-dependent solutions require more complex ways of calculating the RHS wavefunction on the fly. As mentioned, this was accomplished using Qiskit’s Isometry package. An example for creating a wavefunction from an arbitrary vector *U* is as follows:

qc = QuantumCircuit (4)
U = [0.1,2,2,2,2,2,2,0.1]
U /= np.linalg.norm (U)
qc.isometry (U, [0, 1, 2], [])
qc = transpile (qc, basis_gates = [’u3’, ’cx’], optimization_level=3)

**Figure 2:**
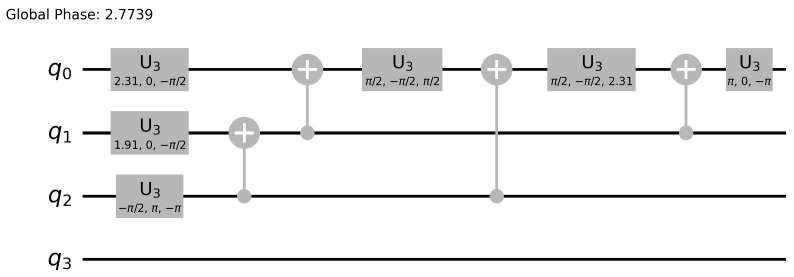
A quantum circuit representing ${\vec{f}}^{T}=\left[\;0.1,2,2,2,2,2,2,0.1\;\right]$ found using Qiskit’s Isometry command.

This circuit is shown in Figure 2.

#### 5.1.1. Poisson Case 1: Parabolic Solution with Homogeneous Boundary Conditions

For the first Poisson test, a manufactured quadratic solution for Equation (5) was used to simplify the RHS preparation. The solution was given by

(9) $$u\left(x\right)=a+b{\left(x-x_{0}\right)}^{2}$$

where $a=g-b{\left(-x_{0}\right)}^{2}$, u(0)=u(1)=g and $x_{0}=1/2$. The RHS of Equation (5) then simplifies to a constant −2b. For homogeneous boundary conditions, where g=0, the reduced RHS of Equation (7) can be written as

(10) $$\begin{array}{cc} \vec{f} & =h{\left[-2b\;\;-2b\;\;-2b\;\;-2b\;\;-2b\;\;-2b\right]}^{T} \end{array}$$

where *h* is the uniform grid spacing and *T* is the transpose.

This linear system was solved using the fixed-ansatz VQLS as described with the Pauli decomposition given in Appendix A and the right-hand side preparation detailed in Section 2.3. For the quantum simulator results without shot noise, errors arose only from discretization of spatial derivatives and the VQLS optimization. The number of qubits *m* in the VQLS determines the grid resolution such that the total number of nodes is $N=2^{m}+2$. Our attempts at optimization for anything greater than three qubits (eight nodes) took too long to simulate on a serial machine. This low qubit count leads to very coarse grids and noticeable discretization errors. To properly converge the discrete problem, finer grids were needed. All ansatz parameters were initialized randomly between $-\pi <=\theta_{k}<=\pi$, default optimizer tolerances of $10^{-4}$ were used, and the initial change to the variables in the COBYLA optimizer was set to rhobeg=π.

**Figure 3:**
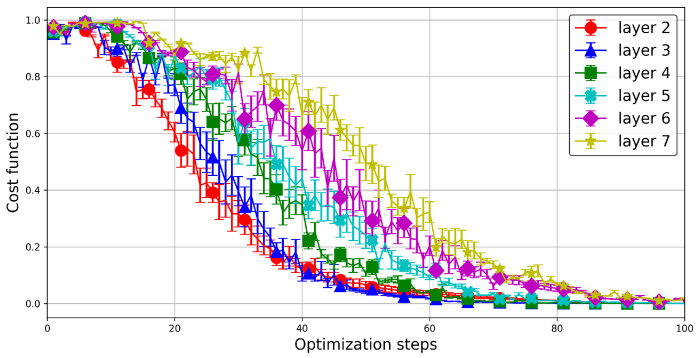
Two-qubit VQLS cost function results for the reduced Poisson problem with homogeneous Dirichlet boundary conditions. The results were averaged over 20 trial runs. Variances are shown by respective bars.

**Figure 4:**
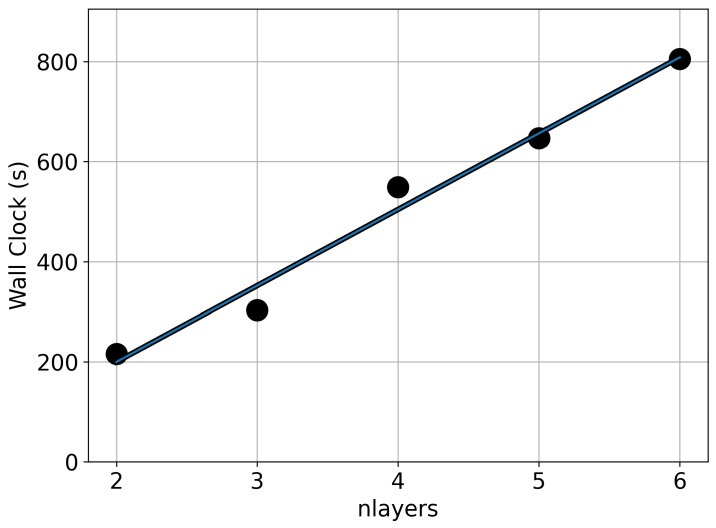
Wall clock time in seconds versus the number of layers for the two-qubit VQLS reduced Poisson problem with homogeneous Dirichlet boundary conditions.

For the two-qubit homogeneous Poisson application, there were four internal and six total finite element nodes. The convergence results for the VQLS for a range of ansatz layers can be seen in Figure 3. These results were averaged over 20 runs, with solid lines indicating the average and variances shown with vertical bars. The two-qubit linear system’s stiffness was a 4×4 matrix. This figure shows that 2 layers were sufficient to successfully capture the solution to the default tolerance within 100 optimization steps. Note that the total number of optimization parameters $N_{\theta }$ varied as $N_{\theta }=m+2\left(m-1\right)\left(nlayers-1\right)$, so for a two-qubit and two-layer network, there were four parameters to span the solution space. This figure shows that as the number of layers or parameters increased, the optimization converged slower, though still relatively fast when compared with the three-qubit problem. This was expected, however, since the solution test space dimensionality was increasing, and the variational algorithm had to span this space. For all layer cases, full solution convergence was achieved within the COBYLA tolerance using the statevector simulator in less than 100 iterations. Figure 4 plots the wall clock time in seconds versus the number of layers averaged over the 20 runs for the 2 qubit problem. From this figure, it is seen that the time it took to converge the solution was linearly proportional to the number of layers used in the variational ansatz.

**Figure 5:**
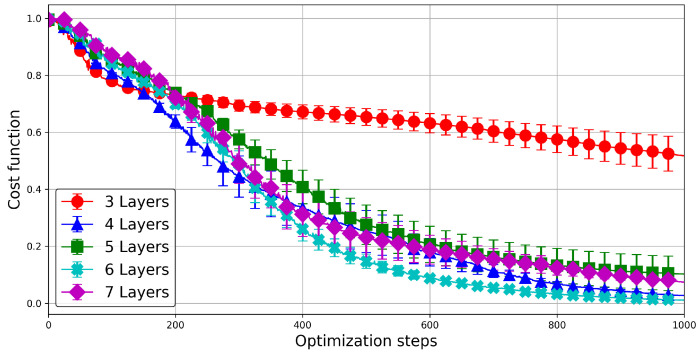
Three-qubit VQLS cost function results for the reduced Poisson problem with homogeneous Dirichlet boundary conditions. The results were averaged over 10 trial runs. Variances are shown by respective bars.

**Figure 6:**
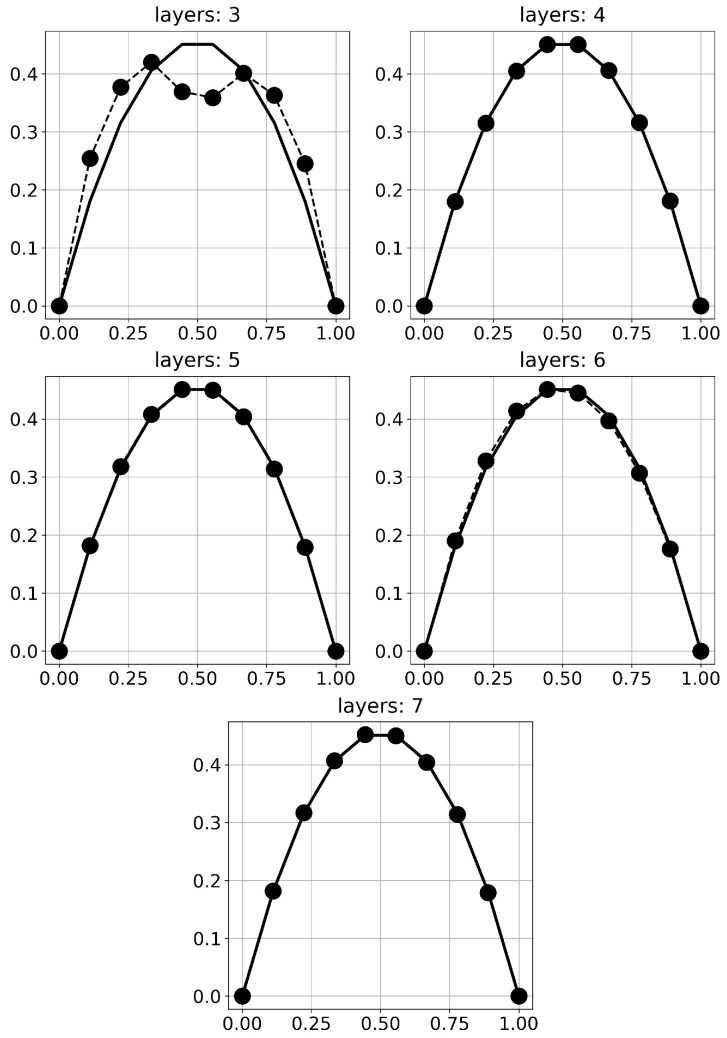
Three-qubit (eight-node) VQLS results (filled circles with dashed lines) for reduced Poisson problem with homogeneous Dirichlet boundary conditions. The classical discrete solution is shown with a solid black line.

**Figure 7:**
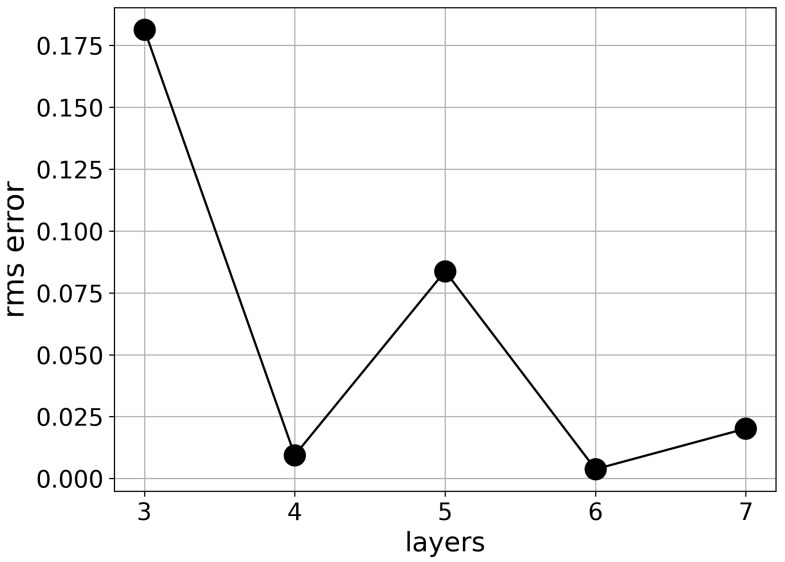
The root mean squared solution error versus the number of layers for the three-qubit VQLS reduced Poisson problem with homogeneous Dirichlet boundary conditions. Here, the errors were averaged over all 10 runs for each layer.

**Figure 8:**
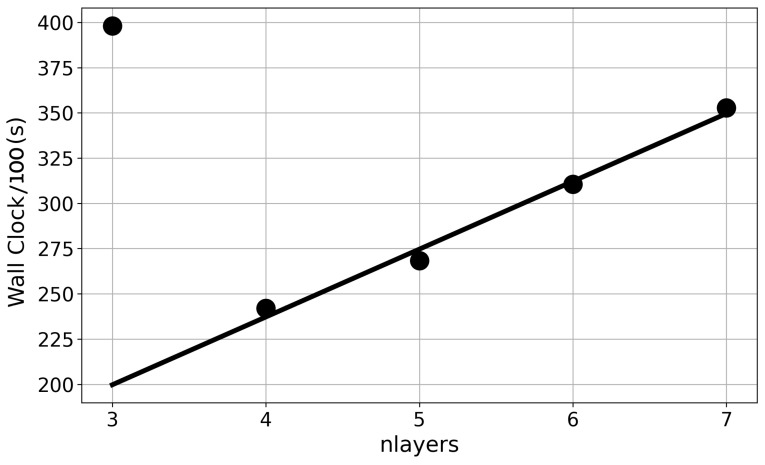
Wall clock time in seconds versus the number of layers for the three-qubit VQLS reduced Poisson problem with homogeneous Dirichlet boundary conditions. The three-layer run does not converge.

Figure 5 displays the cost function of the 3 qubit statevector solution averaged over 10 runs. For this case, there were 8 internal nodes and 10 total, and the linear system stiffness was an 8×8 matrix. While the two-qubit results converged relatively fast for a small number of layers, this was not the case for the three-qubit application. Additionally, it took 4 or more layers for the cost function to converge within 1000 iterations. An interesting note from this figure is that the even-numbered layers performed notably better than the odd layers, with six layers converging in the least amount of time and most accurately. This can be seen more clearly in Figure 6 and Figure 7, which display solution results and the grid root mean square errors averaged over all runs for each layer, respectively. Lastly, Figure 8 displays the time in seconds averaged over all 10 runs for each layer. Since the three-layer run never fully converged within the COBLYA default tolerance, it took the longest. All layers greater than three once again showed a linear increase in time as the layers were incremented.

Figure 6 displays the VQLS versus the classical discrete solution for the three-qubit, eight-internal node problem. In this figure, we see the VQLS solution growing in accuracy as the number of ansatz layers is increased, as expected. In the right column, the VQLS solutions are plotted along with the analytic system solution. Note that the VQLS solution here is being compared to the discrete finite-element solution, and thus both include discretization errors which are not shown.

**Figure 9:**
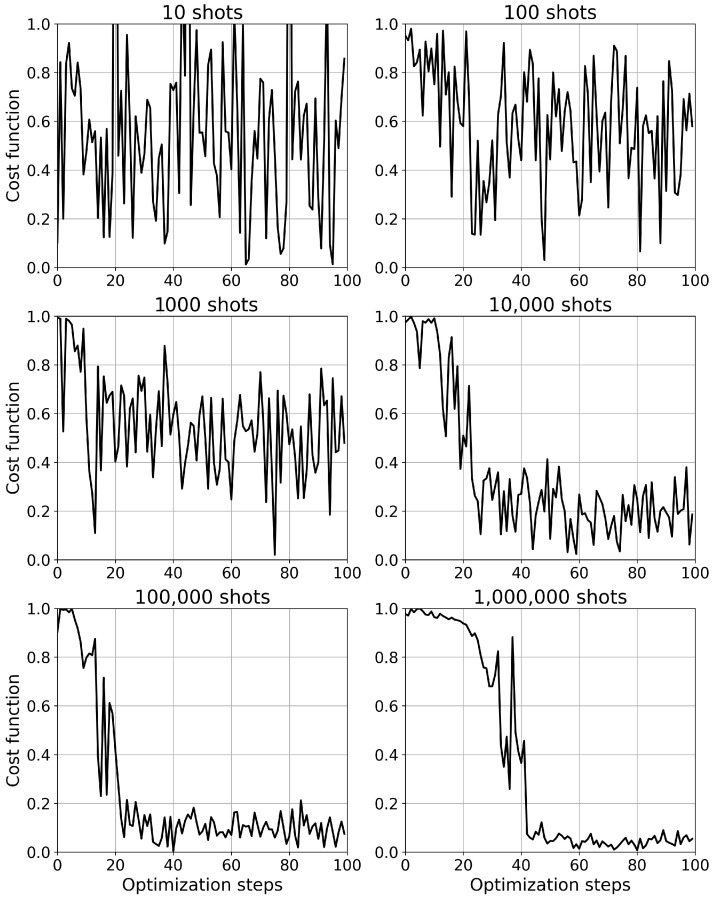
The COBYLA cost convergence for a range of shots in the two-qubit VQLS reduced Poisson problem with homogeneous Dirichlet boundary conditions.

For the VQLS results in Figure 3, Figure 4, Figure 5, Figure 6, Figure 7 and Figure 8, a Qiskit statevector simulator was used so that the full wave function was known, eliminating measurement and sample errors from the convergence figures. For a quantum calculation, however, measurements are necessary, and sampling errors can affect the classical optimizer convergence. Measurements occurred in the Hadamard tests of the cost calculations. Figure 9 shows the COBYLA cost convergence as the number of shots was increased. It was found that to achieve accurate and smooth convergence, at least 100,000 shots were needed for the 2 qubit VQLS system.

#### 5.1.2. Poisson Case 2: Cubic Solution with Non-Homogeneous B.C.

Next, we consider a non-symmetric cubic Poisson solution with non-homogeneous boundaries, which will further complicate the RHS vector as it modifies *f* to be

(11) $$f_{i}=ah^{2}\left(6ih-2\right)+u_{i}\;i=1,n$$

In this case, $u_{i}=u_{L}$ for i=1, and $u_{i}=u_{R}$ for i=n. Note that normally, the RHS addition to the 1D case would be $u_{D}/h$, but both sides are multiplied by *h* in the discrete matrix solution.

**Figure 10:**
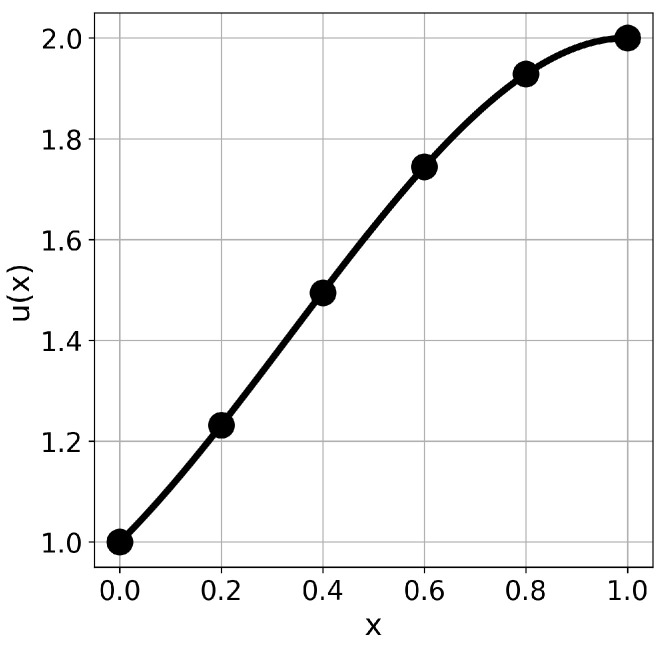
Two-qubit, two-layer solution (filled circles) along with the analytic solution (solid line) of Case 2: the cubic Poisson problem.

**Figure 11:**
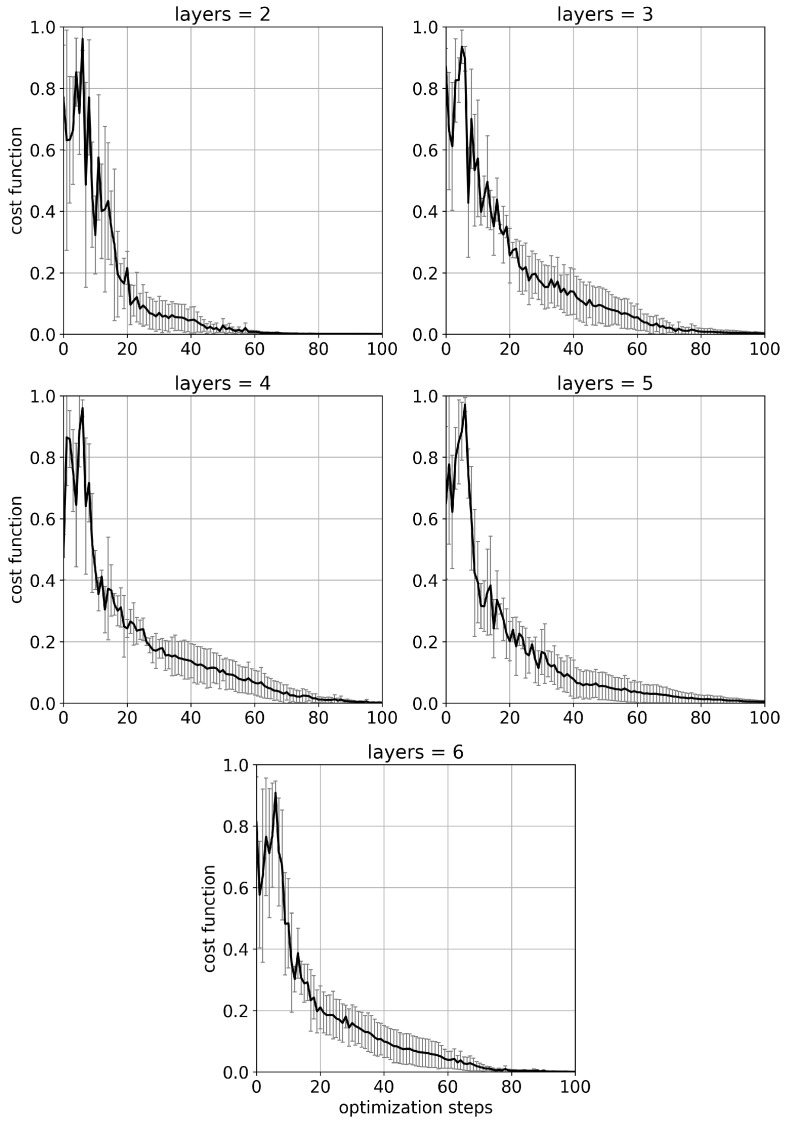
VQLS mean cost versus iteration or optimization count over a range of layers for the cubic Poisson problem. Variances are shown as curve error bars.

The following cubic manufactured solution is used:

(12) $$u\left(x\right)=a\left(-x^{3}+x^{2}+x+1\right)$$

where a=1 so that $u_{L}=1$ and $u_{R}=2$. The two-qubit Qiskit wavefunction simulator was used to calculate the discretized, finite-element VQLS results for 2–6 ansatz layers to investigate the layer count sensitivities to accuracy and convergence. For each layer count, five runs were executed, and the mean and standard deviation of the runs were calculated. Once again, all ansatz parameters were initialized randomly between $-\pi <=\theta_{k}<=\pi$, default tolerances of $10^{-4}$ were used, and the initial change to the variables in the COBYLA optimizer was set to rhobeg=π. Using these parameters, it was found that only two ansatz layers were needed to fully capture the solution, as can be seen in Figure 10. The best cost convergence was also seen for two layers, shown in Figure 11. The cost curve variances did not show any obvious trend with the layer count.

### 5.2. Application 2: The Heat Equation

The Poisson test cases were time-independent and required only one linear solve for the solution. In this section, however, the VQLS results are presented for the 1D time-dependent heat equation

(13) $$\begin{array}{c} \partial_{t}-\partial_{xx}u=0\;x\in \left(0,1\right)\\ u\left(x_{L},t\right)=u_{L}\\ u\left(x_{R},t\right)=u_{R}\\ u\left(x,0\right)=u_{0} \end{array}$$

where $x_{L}$ and $x_{R}$ are the 1D domain endpoints. The weak form of this equation is

(14) $$\int_{x_{L}}^{x_{R}}\partial_{t}u\;\phi \;dx+\int_{x_{L}}^{x_{R}}u_{x}\phi_{x}\;dx=0\;\forall \phi \in H_{0}^{1}\left(0,1\right)$$

Discretizing in time with uniform time steps Δt and using the backward Euler approximation for the time derivative gives

(15) $$\int_{x_{L}}^{x_{R}}u^{k+1}\;\phi \;dx-\int_{x_{L}}^{x_{R}}u^{k}\;\phi \;dx+\Delta t\int_{x_{L}}^{x_{R}}u_{x}^{k+1}\;\phi_{x}\;dx=0$$

where *k* is the discrete time step index such that k=1,nt and nt is the total number of time steps. This equation is made to hold for all test functions, giving the following finite-element (FE) backward Euler matrix equation:

(16) $$\left(M+\Delta tK\right){\vec{u}}^{\;k+1}=M{\vec{u}}^{\;k}$$

For linear basis functions on a uniform grid of a spacing *h*, the matrix operators are

(17) $$M=\left[\begin{array}{ccccc} \frac{2h}{3} & \frac{1h}{6} & 0 & 0 & ....\\ \frac{1h}{6} & \frac{2h}{3} & \frac{1h}{6} & 0 & ...\\ 0 & \frac{1h}{6} & \frac{2h}{3} & \frac{1h}{6} & 0...\\ \vdots & \ddots & \ddots & \ddots & \end{array}\right]$$

(18) $$K=\left[\begin{array}{ccccc} \frac{2}{h} & -\frac{1}{h} & 0 & 0 & .\;...\\ -\frac{1}{h} & \frac{2}{h} & -\frac{1}{h} & 0 & \;...\\ 0 & -\frac{1}{h} & \frac{2}{h} & -\frac{1}{h} & 0\;...\\ \vdots & \ddots & \ddots & \ddots & \end{array}\right]$$

This equation gives a linear system $A\vec{x}=\vec{b}$ at each time step such that

(19) $$A=\left[\begin{array}{ccccc} \frac{2h}{3}+\frac{2\Delta t}{h} & \frac{1h}{6}+\frac{-1\Delta t}{h} & 0 & 0 & ....\\ \frac{1h}{6}+\frac{-1\Delta t}{h} & \frac{2h}{3}+\frac{2\Delta t}{h} & \frac{1h}{6}+\frac{-1\Delta t}{h} & 0 & ...\\ 0 & \frac{1h}{6}+\frac{-1}{h} & \frac{2h}{3}+\frac{2\Delta t}{h} & \frac{1h}{6}+\frac{-1\Delta t}{h} & 0...\\ \vdots & \ddots & \ddots & \ddots & \end{array}\right]$$

and $\vec{b}=M{\vec{u}}^{\;k}$.

**Figure 12:**
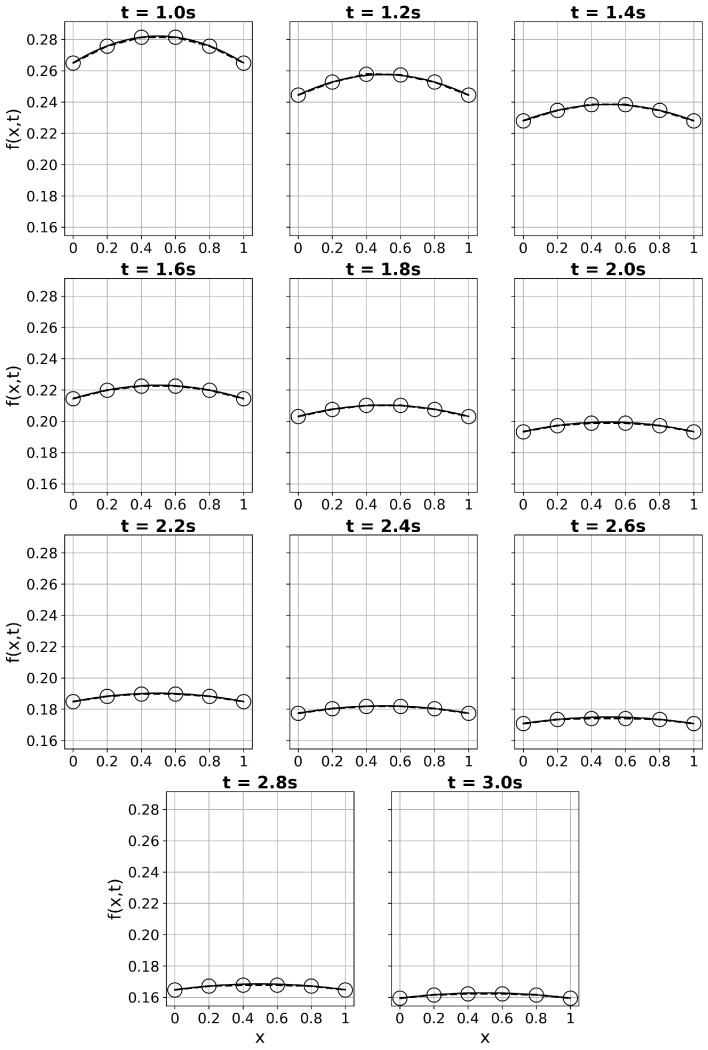
Analytic solution (solid line) versus two-qubit VQLS-based finite element results (dashed line with open circles) for the time-dependent heat equation at each time step.

For verification of the FE-VQLS algorithm, a nonlinear solution was fabricated of the form

(20) $$u\left(x,t\right)=\frac{1}{\sqrt{4\pi t}}exp\left(-\frac{{\left(x-0.5\right)}^{2}}{4t}\right)$$

on the domain [0≤x≤1]×[1≤t≤3]. A uniform grid was created with $n=2^{m}$ internal spatial grid points, N=n+2 total spatial grid points, and nt=11 time points. Figure 12 shows the two-qubit results (dashed lines and open circles) plotted against the analytic solution (solid line). For these results, three layers were used, and *rhobeg* in the COBYLA method was set to π/100.

The results in this figure show excellent agreement between the FE-VQLS and the analytic solution. It should be noted, however, that in order to obtain these results, the FE-VQLS solution had to be scaled appropriately at each time step, since the quantum results were only proportional to the solution. This could be accomplished by using the boundary conditions if they were non-homogeneous, and the system was not solved in a reduced way. However, since the reduced systems were used herein, the non-homogeneous boundaries were not included, and the ratio of the analytic and FE-VQLS solution of the first internal point was used for the scaling.

**Figure 13:**
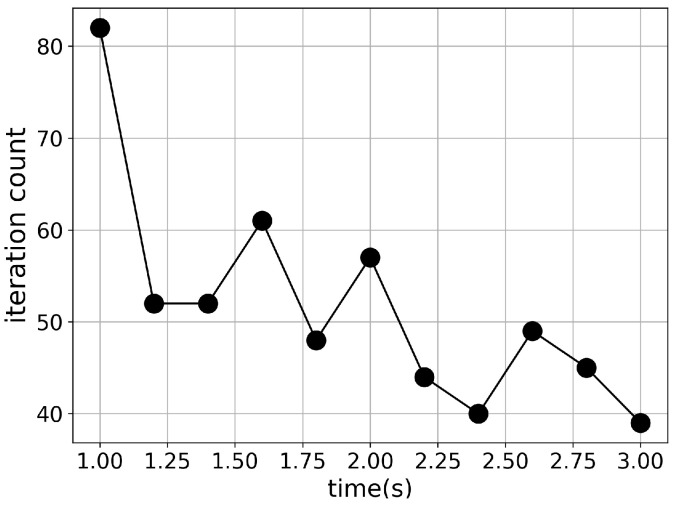
The COBYLA iteration count over time for the two-qubit solution of the heat equation.

At each time step, the previous VQLS ansatz parameters were used to initialize the minimization procedure and speed up convergence. Ideally, the number of COBYLA iterations should decrease in time. This was seen for the two-qubit solution, as shown in Figure 13.

### 5.3. Application 3: The Wave Equation

For the last application, we present the VQLS results for a 1D wave equation of the form

(21) $$\begin{array}{c} \partial_{tt}u-\partial_{xx}u=0\;x\in \left(0,1\right)\\ u\left(x_{L},t\right)=u_{L}\\ u\left(x_{R},t\right)=u_{R}\\ u\left(x,0\right)=u_{0} \end{array}$$

where $x_{L}$ and $x_{R}$ are the endpoints of the 1D domain. The weak form of this equation is

(22) $$\int_{x_{L}}^{x_{R}}\partial_{tt}u\;\phi \;dx+\int_{x_{L}}^{x_{R}}u_{x}\phi_{x}dx=0\;\forall \phi \in H_{0}^{1}\left(0,1\right)$$

Discretizing in time with uniform time steps Δt and using a second-order difference approximation for the time derivative gives

(23) $$\int_{x_{L}}^{x_{R}}u^{k+1}\;\phi \;dx-2\int_{x_{L}}^{x_{R}}u^{k}\;\phi \;dx+\int_{x_{L}}^{x_{R}}u^{k-1}\;\phi \;dx+\Delta t^{2}\int_{x_{L}}^{x_{R}}u_{x}^{k+1}\;\phi_{x}\;dx=0$$

Note here that we have treated the diffusion term implicitly. When applied to all test functions, this yields the matrix equation

(24) $$\left(M+\Delta t^{2}K\right){\vec{u}}^{\;k+1}=M\left(2{\vec{u}}^{\;k}+{\vec{u}}^{\;k-1}\right)$$

where $A=M+\Delta t^{2}K$ and $\vec{b}=M\left(2{\vec{u}}^{\;k}+{\vec{u}}^{\;k-1}\right)$.

To test the VQLS, a non-separable solution of

**Figure 14:**
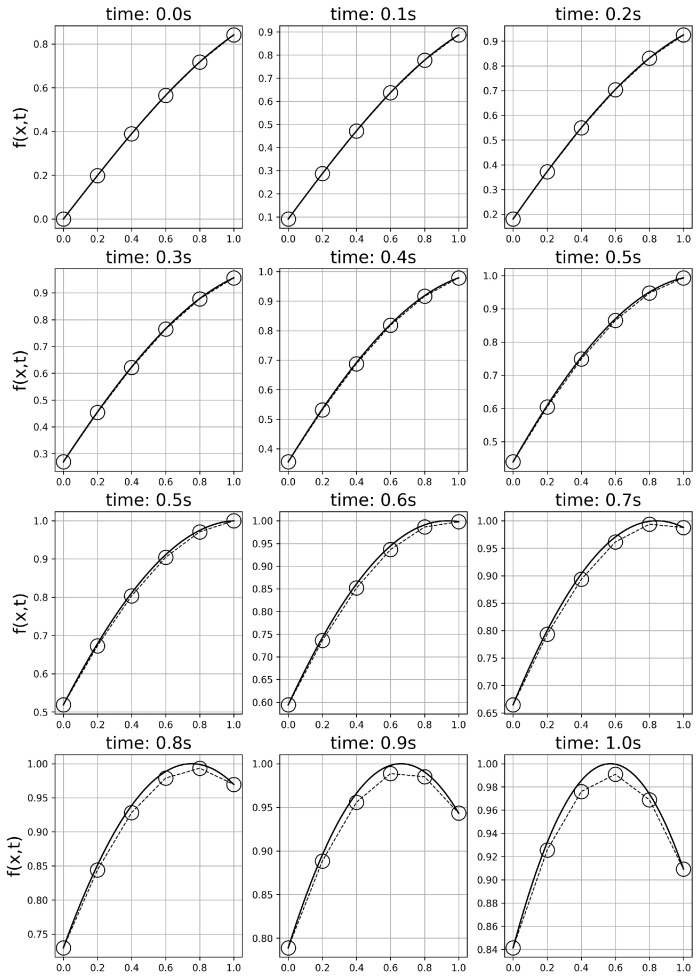
Analytic solution (solid line) versus two-qubit VQLS-based finite element results (dashed line with open circles) for the time-dependent wave equation at each time step.

**Figure 15:**
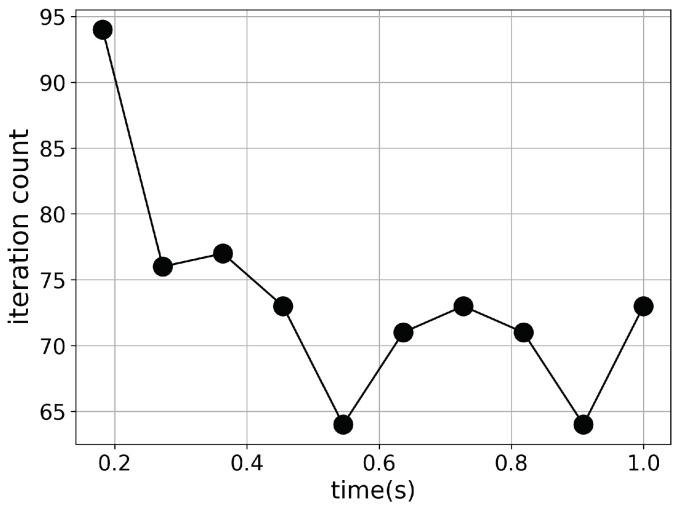
The COBYLA iteration count over time for the two-qubit solution of the wave equation.

(25) u(x,t)=sin(x+t)

was used on the domain [0≤t≤1]×[0≤x≤1]. A total of m=2 qubits (n=4 internal points) were used for the matrix-reduced internal solve with three ansatz layers. The time step was set to Δt=0.1s. As can be seen in Figure 14, the VQLS results agreed well with the analytic solution, and it is noted that a majority of the differences came from discretization and not from the VQLS procedure. The time-dependent COBLYA iteration count, which essentially represents the time evolution regularity of the ansatz parameters, can be seen in Figure 15. This figure shows a large initial iteration count associated with random sampling and a decrease in iteration count for each linear solve as the solution converged over time.

## 6. Discussion

In this study, the variational quantum linear solver recently proposed by Bravo-Prieto et al. [60,61] was used to solve the linear systems obtained from finite-element discretization of the time-independent Poisson and time-dependent heat and wave equations. Although the results presented focused on these equations, the tools of this effort can generally be used to solve any discretization of a partial differential equation that leads to a matrix solution. The key findings of this effort are that (1) the Qiskit Isometry command can be used to generate wavefunctions for arbitrary vectors, a vital component for solving time-dependent right-hand sides, (2) the quantum/classical hybrid variational solver can be used as a potential “accelerator” for discrete finite-element problems, (3) the large number of sampling shots and $N^{2}$ matrix gate Hadamard test evaluation requirements greatly affects qubit scalability and thus the finite element grid resolution, and (4) the minimization iteration count decreases over time as the solution converges, reflecting an ansatz parameter regularity. The latter point is particularly useful for initial value problems, where a set of initial ansatz parameters need only be found once and used thereafter.

Regarding scalability of the VQLS, although it was previously found in [61] that this method was scalable for up to 1024×1024 (10 qubit)-sized systems, that was certainly not the case for the practical linear systems herein, where the matrix and RHS required deeper circuits. Since each term in the stiffness Pauli decomposition requires a Hadamard test against the other terms, this size of the linear combination (circuit depth) directly affects the efficiency of the VQLS algorithm. For the 2 qubit, 6 node systems, the scalability more closely resembled that presented in Bravo-Prieto’s analysis, but the 4 qubit, 18 node systems required 16 Pauli terms in the linear combination, and the VQLS results converged too slowly to be practical.

Future work will include further investigation of (1) new ansatz and optimization options, (2) more efficient methods for creating arbitrary RHS vectors specifically for use in finite-element methods, and (3) the quantum hardware scalability and effect of quantum noise for applications of the FE-VQLS.

## Data Availability

Not applicable.

## Appendix A. Preparation of the Stiffness Matrix

A crucial bottleneck of methods that simulate linear algebra computations with the amplitudes of quantum states is state preparation, which often requires one to initialize a quantum system in a state whose amplitudes reflect the features of the entire dataset. Although efficient methods for state preparation are known for specific cases [66,67], this step easily hides the complexity of the task [68,69].

In order to solve the linear system either directly or variationally on a quantum computer, the stiffness matrix K must be represented as a linear combination of Hermitian unitary operators, $K=\sum_{i}c_{i}\;U_{i}$, representing a system Hamiltonian where $U_{i}$ represents the unitaries and $c_{i}$ represents the complex coefficients. Additionally, we assume that the matrix condition number κ<inf and ∥A∥≤1 and that the $A_{i}$ unitaries can be implemented with efficient quantum circuits. Typically, this decomposition consists of a linear combination of Kronecker products of the Identity and Pauli matrices, as these gates are widely used and recognized. These matrices and gates are defined as follows:

(A1) $$\begin{array}{c} I=\left(\begin{array}{cc} 1 & 0\\ 0 & 1 \end{array}\right),X=\left(\begin{array}{cc} 0 & 1\\ 1 & 0 \end{array}\right),Y=\left(\begin{array}{cc} 0 & -i\\ i & 0 \end{array}\right),Z=\left(\begin{array}{cc} 1 & 0\\ 0 & -1 \end{array}\right) \end{array}$$

To find this linear combination, the tridiagonal matrix can be expressed recursively. Ignoring the diagonal component $I_{2^{m}}$, suppose that we have an expression for the off-diagonal elements in the *m* qubit case

(A2) $$A_{m}=\sum_{i=0}^{2^{m}-2}\left(|i\rangle \langle i+1|+|i+1\rangle \langle i|\right)$$

which gives an expression such as

(A3) $$|0\rangle \langle 1|+|1\rangle \langle 2|+...+|2^{m}-2\rangle \langle 2^{m}-1|+h.c.$$

Now, we write

(A4) $$\begin{array}{cc} I_{2}⊗A_{m} & =\left(|0\rangle \langle 0|+|1\rangle \langle 1|\right)⊗A_{m}\\ \; & =\sum_{i=0}^{2^{m}-2}\left(|i\rangle \langle i+1|+|i+1\rangle \langle i|\right)+\sum_{i=2^{m}}^{2^{m+1}-2}\left(|i\rangle \langle i+1|+|i+1\rangle \langle i|\right)\\ \; & =A_{m+1}-\left(|2^{m}-1\rangle \langle 2^{m}|+|2^{m}\rangle \langle 2^{m}-1|\right) \end{array}$$

and thus

(A5) $$A_{m+1}=I_{2}⊗A_{m}+\left(|2^{m}-1\rangle \langle 2^{m}|+|2^{m}\rangle \langle 2^{m}-1|\right)$$

In the second line of Equation (A4), tensoring |1〉〈1| with the second sum puts a “1” bit ahead of every bit string, which shifts every index in the summand by $2^{m}$. Then, by comparison with Equation (A2), this line is simply $A_{m+1}$ but missing a term connecting the two tri-diagonal block submatrices of $I_{2}⊗A_{m}$.

As an m=3 example, it is first easy to find the off-diagonal solution for m=2, given by

(A6) $$\begin{array}{cc} A_{2} & =I⊗X+\frac{1}{2}\left(X⊗X+Y⊗Y\right)\\ \; & =\left(\begin{array}{cccc} 0 & 1 & 0 & 0\\ 1 & 0 & 0 & 0\\ 0 & 0 & 0 & 1\\ 0 & 0 & 1 & 0 \end{array}\right)+\left(\begin{array}{cccc} 0 & 0 & 0 & 0\\ 0 & 0 & 1 & 0\\ 0 & 1 & 0 & 0\\ 0 & 0 & 0 & 0 \end{array}\right)\\ \; & =\left(\begin{array}{cccc} 0 & 1 & 0 & 0\\ 1 & 0 & 1 & 0\\ 0 & 1 & 0 & 1\\ 0 & 0 & 1 & 0 \end{array}\right) \end{array}$$

Using this result, Equation (A5) can be written as

(A7) $$A_{3}=\left(\begin{array}{cc} A_{2} & 0\\ 0 & A_{2} \end{array}\right)+\left(|011\rangle \langle 100|+|100\rangle \langle 011|\right)$$

### Appendix A.1. Implementing the Recursion Using GHZ States

All that is needed now is an operator representing $\left(|2^{m}-1\rangle \langle 2^{m}|+|2^{m}\rangle \langle 2^{m}-1|\right)$. This turns out to be closely related to writing an m+1 qubit GHZ state in terms of Pauli operators. The GHZ state $|\psi \rangle ={|0\rangle }^{⊗(m+1)}+{|1\rangle }^{⊗(m+1)}$ operator |ψ〉〈ψ| has two off-diagonal elements: one in the top left and one in the top right of the corresponding matrix. These elements can be permuting toward the center of the matrix with the operator

(A8) $$\begin{array}{cc} B_{m+1} & =|0\rangle \langle 2^{m+1}-1|+|2^{m+1}-1\rangle \langle 0|\\ \; & ={\left(|0\rangle \langle 1|\right)}^{⊗(m+1)}+{\left(|1\rangle \langle 0|\right)}^{⊗(m+1)} \end{array}$$

Thus, we have

(A9) $$\begin{array}{cc} \left(X⊗I_{2^{m}}\right)B_{m+1}\left(X⊗I_{2^{m}}\right) & =\left(X⊗I_{2^{m}}\right)\left({\left(|0\rangle \langle 1|\right)}^{⊗(m+1)}+{\left(|1\rangle \langle 0|\right)}^{⊗(m+1)}\right)\left(X⊗I_{2^{m}}\right)\\ \; & =(|10...0\rangle \langle 01...1|+|01...1\rangle \langle 10...0|)\\ \; & =|2^{m}\rangle \langle 2^{m}-1|+|2^{m}-1\rangle \langle 2^{m}| \end{array}$$

Now, using the results of (GUHNE 2007), the center shift operator can be written as

(A10) $$\begin{array}{cc} B_{m} & ={\left(|0\rangle \langle 1|\right)}^{⊗m}+{\left(|1\rangle \langle 0|\right)}^{⊗m} \end{array}$$

(A11) $$\begin{array}{cc} \; & =\frac{1}{2^{m-1}}\sum_{t=0}^{\left\lfloor m/2\right\rfloor }{\left(-1\right)}^{t}\sum_{\pi }S_{\pi }\left(X^{⊗(m=2t)}⊗Y^{⊗2t}\right) \end{array}$$

Here, the $S_{\pi }$ operator permutes *m* subsystems according to a permutation π:{1,⋯,n}→{1,⋯,m}, and the sum runs over all unique permutations π on size *m* sets. Using this formula along with Equation (A5) gives an analytic Pauli decomposition of the stiffness matrix.

### Appendix A.2. Preparation of the m=3 Stiffness Matrix

For the m=3 qubit stiffness matrix (with $2^{m}=8$ finite element nodes), Equation (A10) becomes

(A12) $$B_{3}=\frac{1}{4}\left(\mathrm{XXX}-\mathrm{XYY}-\mathrm{YXY}-\mathrm{YYX}\right)$$

and therefore

(A13) $$\left(X⊗I_{4}\right)B_{3}\left(X⊗I_{4}\right)\;=\frac{1}{4}\left(\mathrm{XXX}-\mathrm{XYY}+\mathrm{YXY}+\mathrm{YYX}\right)$$

Substituting this into Equation (A5) and adding the diagonal factor 2III gives our final three-qubit, eight-node stiffness matrix Pauli decomposition as follows:

(A14) $$\begin{array}{cc} 2I_{8}-A_{3} & =2I_{8}-\left[I_{2}⊗A_{2}+\left(X⊗I_{4}\right)B_{3}\left(X⊗I_{4}\right)\right]\\ \; & =2\mathrm{III}-\left[\mathrm{IIX}+\frac{1}{2}\left(\mathrm{IXX}+\mathrm{IYY}\right)+\frac{1}{4}\left(\mathrm{XXX}-\mathrm{XYY}+\mathrm{YXY}+\mathrm{YYX}\right)\right] \end{array}$$

### Appendix A.3. Preparation of a General m Qubit Stiffness Matrix

Generalization of the above procedure for an *m* qubit stiffness matrix gives the following recursive procedure with $A_{1}:=x$:

(A15) $$\begin{array}{cc} A_{m} & =I_{2}⊗A_{m-1}+\\ \; & \left(X⊗I_{2^{m-1}}\right)\frac{1}{2^{m-1}}\sum_{t=0}^{\left\lfloor m/2\right\rfloor }{\left(-1\right)}^{t}\sum_{\pi }S_{\pi }\left(X^{⊗(m=2t)}⊗Y^{⊗2t}\right)+\left(X⊗I_{2^{m-1}}\right) \end{array}$$

The final finite-element stiffness matrix is then

(A16) $$K_{m}=2I_{2^{m}}-A_{m}$$

## References

[1] Preskill J. (2012). Quantum computing and the entanglement frontier. arXiv.

[2] Harrow A.W., Montanaro A. (2017). Quantum computational supremacy. Nature.

[3] Arute F., Arya K., Babbush R., Bacon D., Bardin J.C., Barends R., Biswas R., Boixo S., Brandao F.G.S.L., Buell D.A. (2019). Quantum supremacy using a programmable superconducting processor. Nature.

[4] Connor E. (2020). The New Light-Based Quantum Computer Jiuzhang Has Achieved Quantum Supremacy. https://www.sciencenews.org/article/new-light-based-quantum-computer-jiuzhang-supremacy.

[5] Head-Marsden K., Flick J., Ciccarino C.J., Narang P. (2021). Quantum information and algorithms for correlated quantum matter. Chem. Rev..

[6] Breuer H.-P., Petruccione F. (2007). The Theory of Open Quantum Systems.

[7] Clerk A.A., Devoret M.H., Girvin S.M., Marquardt F., Schoelkopf R.J. (2010). Introduction to quantum noise, measurement, and amplification. Rev. Mod. Phys..

[8] Lidar D.A. (2019). Lecture notes on the theory of open quantum systems. arXiv.

[9] Krantz P., Kjaergaard M., Yan F., Orlando T.P., Gustavsson S., Oliver W.D. (2019). A quantum engineer’s guide to superconducting qubits. Appl. Phys. Rev..

[10] Kandala A., Temme K., Córcoles A.D., Mezzacapo A., Chow J.M., Gambetta J.M. (2019). Error mitigation extends the computational reach of a noisy quantum processor. Nature.

[11] McArdle S., Yuan X., Benjamin S. (2019). Error-mitigated digital quantum simulation. Phys. Rev. Lett..

[12] Smart S.E., Mazziotti D.A. (2019). Quantum-classical hybrid algorithm using an error-mitigating n -representability condition to compute the mott metal-insulator transition. Phys. Rev. A.

[13] Smart S.E., Boyn J.N., Mazziotti D.A. (2022). Resolving correlated states of benzyne with an error-mitigated contracted quantum eigensolver. Phys. Rev. A.

[14] Endo S., Cai Z., Benjamin S.C., Yuan X. (2021). Hybrid quantum-classical algorithms and quantum error mitigation. J. Phys. Soc. Jpn..

[15] Smart S.E., Hu Z., Kais S., Mazziotti D.A. (2022). Relaxation of stationary states on a quantum computer yields a unique spectroscopic fingerprint of the computer’s noise. Commun. Phys..

[16] Aleksandrowicz G., Alexander T., Barkoutsosa P., Bello L., Ben-Haim Y., Bucher D., Cabrera-Hernández F., Carballo-Franquis J., Chen A., Chen C. (2019). Qiskit: An Open-Source Framework for Quantum Computing.

[17] Amazon, Amazon Braket. https://aws.amazon.com/braket/.

[18] IBM, Learning Quantum Computation Using Qiskit. http://qiskit.org/textbook.

[19] Albornoz C., Alonso G., Andrenkov P.A.M., Asadi A. (2021). Anothers, Xanadu Quantum Codebook. https://codebook.xanadu.ai.

[20] Qbraid Qbraid: Cloud-Based ide for Quantum Computing. https://qbraid.com.

[21] Biamonte J., Wittek P., Pancotti N., Rebentrost P., Wiebe N., Lloyd S. (2017). Quantum machine learning. Nature.

[22] Pudenz K.L., Lidar D.A. (2013). Quantum adiabatic machine learning. Quantum Inf. Process..

[23] Lloyd S., Mohseni M., Rebentrost P. (2014). Quantum principal component analysis. Nat. Phys..

[24] Rebentrost P., Mohseni M., Lloyd S. (2014). Quantum support vector machine for big data classification. Phys. Rev. Lett..

[25] Schuld M., Sinayskiy I., Petruccione F. (2015). An introduction to quantum machine learning. Contemp. Phys..

[26] Altaisky M.V., Zolnikova N.N., Kaputkina N.E., Krylov V.A., Lozovik Y.E., Dattani N.S. (2016). Towards a feasible implementation of quantum neural networks using quantum dots. Appl. Phys. Lett..

[27] Dunjko V., Taylor J.M., Briegel H.J. (2015). Framework for learning agents in quantum environments. arXiv.

[28] Alvarez-Rodriguez U., Lamata L., Escandell-Montero P., Martín-Guerrero J.D., Solano E. (2017). Supervised quantum learning without measurements. Sci. Rep..

[29] Lamata L. (2017). Basic protocols in quantum reinforcement learning with superconducting circuits. Sci. Rep..

[30] Wiebe N., Braun D., Lloyd S. (2012). Quantum algorithm for data fitting. Phys. Rev. Lett..

[31] Schuld M., Sinayskiy I., Petruccione F. (2016). Prediction by linear regression on a quantum computer. Phys. Rev. A.

[32] Harrow A.W., Hassidim A., Lloyd S. (2009). Quantum algorithm for linear systems of equations. Phys. Rev. Lett..

[33] Berry D.W., Childs A.M., Kothari R. Hamiltonian simulation with nearly optimal dependence on all parameters. Proceedings of the 2015 IEEE 56th Annual Symposium on Foundations of Computer Science.

[34] Zhao Z., Fitzsimons J.K., Fitzsimons J.F. (2019). Quantum-assisted Gaussian process regression. Phys. Rev. A.

[35] Zheng Y., Song C., Chen M.C., Xia B., Liu W., Guo Q., Zhang L., Xu D., Deng H., Huang K. (2017). Solving systems of linear equations with a superconducting quantum processor. Phys. Rev. Lett..

[36] Lee Y., Joo J., Lee S. (2019). Hybrid quantum linear equation algorithm and its experimental test on ibm quantum experience. Sci. Rep..

[37] Pan J., Cao Y., Yao X., Li Z., Ju C., Chen H., Peng X., Kais S., Du J. (2014). Experimental realization of quantum algorithm for solving linear systems of equations. Phys. Rev. A.

[38] Cai X.-D., Weedbrook C., Su Z.-E., Chen M.-C., Gu M., Zhu M.-J., Li L., Liu N., Lu C., Pan J. (2013). Experimental quantum computing to solve systems of linear equations. Phys. Rev. Lett..

[39] Barz S., Kassal I., Ringbauer M., Lipp Y.O., Dakić B., Aspuru-Guzik A., Walther P. (2014). A two-qubit photonic quantum processor and its application to solving systems of linear equations. Sci. Rep..

[40] Wen J., Kong X., Wei S., Wang B., Xin T., Long G. (2019). Experimental realization of quantum algorithms for a linear system inspired by adiabatic quantum computing. Phys. Rev. A.

[41] Anschuetz E., Olson J., Aspuru-Guzik A., Cao Y., Feld S., Linnhoff-Popien C. (2019). Variational quantum factoring. Quantum Technology and Optimization Problems.

[42] Peruzzo A., McClean J., Shadbolt P., Yung M., Zhou X., Love P.J., Aspuru-Guzik A., O’Brien J.L. (2014). A variational eigenvalue solver on a photonic quantum processor. Nat. Commun..

[43] Cao Y., Romero J., Olson J.P., Degroote M., Johnson P.D., Kieferova M., Kivlichan I.D., Menke T., Peropadre B., Sawaya N.P.D. (2019). Quantum chemistry in the age of quantum computing. Chem. Rev..

[44] Higgott O., Wang D., Brierley S. (2019). Variational quantum computation of excited states. Quantum.

[45] Jones T., Endo S., McArdle S., Yuan X., Benjamin S.C. (2019). Variational quantum algorithms for discovering hamiltonian spectra. Phys. Rev. A.

[46] Li Y., Benjamin S.C. (2017). Efficient variational quantum simulator incorporating active error minimization. Phys. Rev. X.

[47] Kokail C., Maier C., van Bijnen R., Brydges T., Joshi M.K., Jurcevic P., Muschik C.A., Silvi P., Blatt R., Roos C.F. (2019). Self-verifying variational quantum simulation of lattice models. Nature.

[48] Heya K., Nakanishi K.M., Mitarai K., Fujii K. (2019). Subspace variational quantum simulator. arXiv.

[49] Cirstoiu C., Holmes Z., Iosue J., Cincio L., Coles P.J., Sornborger A. (2020). Variational fast forwarding for quantum simulation beyond the coherence time. npj Quantum Inf..

[50] Yuan X., Endo S., Zhao Q., Li Y., Benjamin S.C. (2019). Theory of variational quantum simulation. Quantum.

[51] Romero J., Olson J.P., Aspuru-Guzik A. (2017). Quantum autoencoders for efficient compression of quantum data. Quantum Sci. Technol..

[52] LaRose R., Tikku A., O’Neel-Judy É., Cincio L., Coles P.J. (2019). Variational quantum state diagonalization. npj Quantum Inf..

[53] Bravo-Prieto C., Garcí a-Martín D., Latorre J.I. (2020). Quantum singular value decomposer. Phys. Rev. A.

[54] Cerezo M., Sharma K., Arrasmith A., Coles P.J. (2022). Variational quantum state eigensolver. npj Quantum Inf..

[55] Khatri S., LaRose R., Poremba A., Cincio L., Sornborger A.T., Coles P.J. (2019). Quantum-assisted quantum compiling. Quantum.

[56] Jones T., Benjamin S.C. (2022). Robust quantum compilation and circuit optimisation via energy minimisation. Quantum.

[57] Arrasmith A., Cincio L., Sornborger A.T., Zurek W.H., Coles P.J. (2019). Variational consistent histories as a hybrid algorithm for quantum foundations. Nat. Commun..

[58] Cerezo M., Poremba A., Cincio L., Coles P.J. (2020). Variational quantum fidelity estimation. Quantum.

[59] Koczor B., Endo S., Jones T., Matsuzaki Y., Benjamin S. (2020). Variational-state quantum metrology. New J. Phys..

[60] Bravo-Prieto C., LaRose R., Cerezo M., Subasi Y., Cincio L., Coles P.J. (2019). Variational Quantum Linear Solver. arXiv.

[61] Bravo-Prieto C., LaRose R., Cerezo M., Subaşı Y., Cincio L., Coles P.J. (2020). Variational quantum linear solver: A hybrid algorithm for linear systems. Bull. Am. Phys. Soc..

[62] Cincio L., Subaşı Y., Sornborger A.T., Coles P.J. (2018). Learning the quantum algorithm for state overlap. New J. Phys..

[63] Pesce R.M.N., Stevenson P.D. (2021). H2zixy: Pauli spin matrix decomposition of real symmetric matrices. arXiv.

[64] Pellow-Jarman A., Sinayskiy I., Pillay A., Petruccione F. (2021). A comparison of various classical optimizers for a variational quantum linear solver. Quantum Inf. Process..

[65] Hughes T.J. (2012). The Finite Element Method: Linear Static and Dynamic Finite Element Analysis.

[66] Soklakov A.N., Schack R. (2006). Efficient state preparation for a register of quantum bits. Phys. Rev. A.

[67] Giovannetti V., Lloyd S., Maccone L. (2008). Quantum Random Access Memory. Phys. Rev. Lett..

[68] Aaronson S. (2015). Read the fine print. Nat. Phys..

[69] Bang J., Dutta A., Lee S.W., Kim J. (2019). Optimal usage of quantum random access memory in quantum machine learning. Phys. Rev. A.

